# Norovirus Vaccines: Current Clinical Development and Challenges

**DOI:** 10.3390/pathogens10121641

**Published:** 2021-12-19

**Authors:** Ming Tan

**Affiliations:** 1 Division of Infectious Diseases, Cincinnati Children’s Hospital Medical Center, Cincinnati, OH 45229, USA; ming.tan@cchmc.org; 2 Department of Pediatrics, University of Cincinnati College of Medicine, Cincinnati, OH 45229, USA

**Keywords:** norovirus, gastroenteritis, vaccine, immunogenicity, antiviral

## Abstract

Noroviruses are the major viral pathogens causing epidemic and endemic acute gastroenteritis with significant morbidity and mortality. While vaccines against norovirus diseases have been shown to be of high significance, the development of a broadly effective norovirus vaccine remains difficult, owing to the wide genetic and antigenic diversity of noroviruses with multiple co-circulated variants of various genotypes. In addition, the absence of a robust cell culture system, an efficient animal model, and reliable immune markers of norovirus protection for vaccine evaluation further hinders the developmental process. Among the vaccine candidates that are currently under clinical studies, recombinant VP1-based virus-like particles (VLPs) that mimic major antigenic features of noroviruses are the common ones, with proven safety, immunogenicity, and protective efficacy, supporting a high success likelihood of a useful norovirus vaccine. This short article reviews the recent progress in norovirus vaccine development, focusing on those from recent clinical studies, as well as summarizes the barriers that are being encountered in this developmental process and discusses issues of future perspective.

## 1. Introduction

Noroviruses, members of the *Norovirus* genus in the family *Caliciviridae*, are a group of nonenveloped, single-stranded, positive-sense RNA viruses. They are the most common viral pathogens causing epidemic and endemic acute gastroenteritis (AGE) with typical symptoms of watery diarrhea, vomiting, and stomach cramping, affecting humans in all age groups in both developing and developed countries. Noroviruses are highly contagious with a reproductive number of Ro > 2 [1]. They spread quickly through polluted water, food, and/or surfaces, often leading to large AGE outbreaks in a variety of closed and semi-closed settings, including long-term care facilities, schools, hospitals, military installations, and cruise ships. In addition, noroviruses are commonly responsible for community-acquired, sporadic diarrhea cases. It was estimated that norovirus infections cause approximately 700 million episodes of diarrhea globally, accounting for 20% of all diarrhea cases annually, with substantial morbidity and mortality [2]. In the U.S.A. alone, noroviruses cause around 20 million AGE incidents every year, leading to about 70,000 hospitalizations, and up to 800 deaths [3,4]. On a worldwide basis, the biggest norovirus disease burden occurs in low- and middle-income countries, claiming over 200,000 lives per annum with USD 4.2 billion in direct health system costs and USD 60.3 billion in social economic loss [5,6]. Thus, noroviruses represent a major global public health threat. 

Since 2016, the WHO has recognized the development of a norovirus vaccine as a high priority [7]. However, the development of a broadly effective norovirus vaccine has been difficult, being affected by a few major factors. First, human noroviruses are genetically and antigenically diverse, consisting of five genogroups (GI, GII, GIV, GVIII, and GIX) which comprise 35 genotypes [8]. Multiple variants of various genotypes are often detected simultaneously. Even within a single genotype, such as the predominant genogroup II, genotype 4 (GII.4), new variants emerge periodically as a result of rapid evolution [9,10,11]. Second, noroviruses do not effectively grow in culture cells, making the conventional live attenuated or inactivated vaccine approaches unfeasible. Consequently, the nonreplicating, recombinant protein-based vaccine strategy, mostly using virus-like particles (VLPs), becomes a common choice. Third, the unavailability of a standard cell culture-based neutralization assay, an efficient animal model, and trustworthy immune corelates of norovirus protective immunity to evaluate vaccine candidates add further obstacles in the vaccine development. Despite these drawbacks, advancements have been achieved, with four candidate vaccines having reached the stage of clinical development. While several review papers were published recently, giving systematic overviews on the preclinical developments of various norovirus vaccine candidates [12,13,14,15,16,17], this short article focuses on the current advances in clinical evaluations of four vaccine candidates.

## 2. Norovirus Vaccine Candidates under Clinical Trials

### 2.1. The Takeda Norovirus Vaccine

Among the norovirus vaccine candidates that were in clinical research (Table 1), the TAK-214 developed by Takeda Pharmaceuticals International AG [18] is the most studied one. This is an adjuvanted VLP-based bivalent vaccine containing two types of VLPs, one from the GI.1 Norwalk virus (NV) strain and the other one from a consensus GII.4 sequence (GII.4c) derived from three GII.4 variants, including Yerseke/2006a, Den Haag/2006b, and Houston/2002 variants. GII.4 represents the predominant genotype that causes most norovirus disease burden globally [19]. The inclusion of both GI and GII norovirus antigens intends to broaden its potential protective immunity. Several phase 1 clinical studies were conducted, proving good safety and tolerability, as well as vigorous immune responses to both antigen components of the vaccine in different formulations in healthy adults [20,21,22,23]. A further one-year follow-up study with memory probe vaccination showed persistence of serum antibodies to the two VLP components of the vaccine [23].

Following the phase 1 studies, three phase 2 clinical trials were performed to assess the efficacy of the Takeda’s vaccine candidate. In a phase 2a trial, two doses of intranasally delivered vaccine using chitosan and monophosphoryl lipid A (MPLA) as adjuvants were demonstrated to protect healthy adults against challenge of homologous GI.1 NV [24]. In total, 70% of the vaccine recipients revealed an NV-specific IgG seroresponse that was defined as four-fold rise in serum antibody titers. Vaccination significantly decreased incidences of AGE (*p* = 0.006) and viral infection (*p* = 0.05) caused by NV challenge. In another phase 2a study [25], the bivalent vaccine adjuvanted with MPLA and aluminum salt was administered intramuscularly for two doses, which reduced vomiting and/or diarrhea caused by challenge with a GII.4 Farmington Hills/2002 variant (*p* = 0.054). These results collectively indicated the protective immunity conferred by the Takeda’s vaccine against AGE diseases caused by the two homologous norovirus strains.

The first in-human phase 2b field efficacy study of the TAK-214 vaccine was conducted over two winter seasons in a U.S. Navy training facility with over 4600 participants [26]. The outcomes showed a vaccine effectiveness of 61.8% (95.01% CI, 20.8 to 81.6; *p* = 0.0097) against moderate to severe AGE caused by noroviruses of different genotypes, including GI.1 (*n* = 1), G1.7 (*n* = 1), GII.2 (*n* = 39), and GII.4 (*n* = 7). What is noteworthy is that the vaccine efficacy against GII.2 noroviruses was 57.4% (95% CI, 7.0 to 80.5; *p* = 0.0321), indicating that the TAK-214 vaccine elicits a broad immune response and confers cross-genotype protection, although such protection might be due to previous exposures of the study participants to GII.2 or similar noroviruses [26]. It was also noted that serum samples of the vaccinees and some placebo recipients who were infected by GII.2 noroviruses with AGE revealed increased titers of blocking antibodies against GI.1/GII.4 VLPs attaching to histo-blood group antigens (HBGAs). HBGAs are host attachment factors or receptors for norovirus infection [27,28,29]. The HBGA blocking antibodies were found to be associated with protection against norovirus-induced AGE [30,31]. These data suggested that TAK-214 vaccine, and likely also other norovirus vaccines, could induce certain cross-genotype protective immunity.

### 2.2. The Vaxart Norovirus Vaccine

Another norovirus vaccine candidate in clinical study is a recombinant VP1-based bivalent vaccine (VXA-NVV-104) [32] created by Vaxart Pharmaceutical Inc. based on its proprietary oral tablet technology (Table 1) [33]. This vaccine contains recombinant adenovirus-based vectors [34] carrying genes encoding noroviral VP1s to express VP1 antigens locally in the epithelial cells within the intestine of vaccine recipients to induce mucosal immunity. The adenovirus vector also contains specific RNA-encoding sequences to generate double-stranded RNAs as an adjuvant for enhanced immunogenicity of the VP1 antigens. Both VP1-encoding genes of the GI.1 NV strain and the GII.4 Sydney variant are included as a bivalent vaccine for broad efficacy. Therefore, this tablet vaccine is designed to be administered orally to elicit the mucosal immune response locally within the intestine to prevent norovirus infection. Although direct evidence remains lacking, it is plausible to expect that the adenovirus-expressing VP1s self-assemble into noroviral VLPs in the epithelial cells of the intestine of vaccine recipients, because noroviral VP1s have a high propensity to form VLPs spontaneously and the epithelial cells of the intestine are the natural host cells of noroviruses.

An early monovalent formulation of the vaccine expressing the VP1 antigen of the GI.1 NV strain (VXA-G1.1-NN) was studied by a phase 1 clinical trial [33]. The results showed that this oral vaccine was well tolerated. After a single dose immunization, vaccine recipients elicited significantly higher HBGA-blocking antibody titers (*p* = 0.0003). The vaccinees also generated mucosally primed NV-specific circulating antibody-secreting cells (ASCs), IgA+ memory B cells with gut-homing receptor (α4β7) and fecal IgA. These data indicated substantial systemic and mucosal immune responses that are potentially relevant to protective immunity against norovirus infection, supporting the notion that this oral tablet vaccine is a promising one. Recently, Vaxart announced a new phase 1b norovirus dose-ranging trial in elderly adults [32], which will evaluate the safety, tolerability, immunogenicity, and efficacy of the vaccine with a two-dose vaccination schedule in healthy older adults between 55 and 80 years old (ClinicalTrials.gov identifier: NCT04854746). The study is currently recruiting participants and is estimated to be completed by 1 June 2022.

### 2.3. The NVSI Norovirus Vaccine

The third norovirus vaccine candidate under clinical study is a bivalent VLP-based vaccine (Hansenulapolymorpha) that was developed by the National Vaccine and Serum Institute (NVSI) of China (ClinicalTrials.gov identifier: NCT04188691, Table 1). It is composed of two recombinant VLPs representing the GI.1 and the GII.4 genotypes, respectively, with aluminum salt as the adjuvant. A phase 1 trial to evaluate the safety and immunogenicity of the vaccine was just completed in which 510 healthy subjects aged between 6 months and 59 years were enrolled. Two or three doses of the vaccine were administered intramuscularly. Outcome measures included adverse effects after immunization, GI.1 and GII.4 norovirus specific IgG antibody titers and their positive rates, as well as GI.1 and GII.4 norovirus HBGA-blocking antibody titers and their positive rates. Based on the schedule (ClinicalTrials.gov identifier: NCT04188691), this study was completed by 13 December 2020, but the results are not yet published in a scientific journal.

### 2.4. The Longkoma Norovirus Vaccine

This is a quadrivalent vaccine consisting of four aluminum salt adjuvanted VP1 proteins, each representing GI.1, GII.3, GII.4, or GII.17 genotypes, produced by means of a yeast expression system (ClinicalTrials.gov identifier: NCT04563533). The vaccine was developed by the Institut Pasteur of Shanghai (IPS) (Shanghai, China) and Anhui Zhifei Longcom Biopharmaceutical Co. Ltd. (Hefei, China) in China. Phase 1 and phase 2a clinical trials were registered (ClinicalTrials.gov identifier: NCT04563533), aiming to (1) assess the safety and tolerability of the vaccine at various doses, (2) evaluate the vaccine immune response, and (3) determine the optimal vaccine dose for future development. In total, 580 subjects aged between 6 weeks and >60 years will be enrolled and sorted into 5 age groups, categorized as infants, toddlers, adolescents, adults, and elderly. The vaccine will be administered intramuscularly, and outcome measures will include adverse events, positive conversion rates in a four-fold increase of norovirus-specific IgA, IgG, and HBGA blocking antibodies after immunization. These clinical studies started on 5 August 2020 and are expected to be completed by 7 July 2022. Data from these studies will further help our understanding of immune responses of norovirus vaccines in humans, particularly in infants and young children. It should be noted that the data of Hansenulapolymorpha and the Longkoma vaccines are summarized from Clinicaltrial.gov webpages (assessed on 18 December 2021); these data have not yet been peer-reviewed for formal publication.

## 3. Major Challenges to Develop a Broadly Effective Norovirus Vaccine and Future Perspective

Several major barriers were noted during the development of a norovirus vaccine. Noroviruses are known for their wide genetic and antigenic diversity. Infection by a single norovirus strain does not appear to confer a broad, long-lasting immunity [35]. The cross protection observed during the phase 2b field study of Takada’s vaccine may be due to previous exposures of the subjects to those genotypes [26]. Among the four norovirus vaccine candidates under clinical trials, the three bivalent vaccines contain similar antigen components of a GI.1 and a GII.4 strain (Table 1). These selections of antigens are based on the rationales that the predominant GII.4 genotype is responsible for most of the AGE burden of noroviruses [10] and that an inclusion of a GI strain could broaden the vaccine effectiveness because a previous study of a cocktail VLP vaccine candidate showed rapid rises in antibodies that blocked against norovirus VLPs of diverse genotypes binding to HBGA ligands [36]. On the other hand, since other non-GII.4 genotypes, such as GII.3 and GII.17, often contributed substantially to norovirus disease burden globally in the past [37], the Longkoma vaccine containing VP1 antigens of all four genotypes should provide a wider breadth of effectiveness compared with the bivalent vaccine candidates and, thus, may represent a future direction of norovirus vaccine development. In fact, GII.2 noroviruses were found to be predominant between 2016 and 2019 in China [37,38,39], suggesting that an even more complex panel of antigens representing more norovirus genotypes should be considered for even broader vaccine efficacy.

Regarding the longevity of norovirus immune protection, limited studies showed that a natural infection resulted in protective immunity against homologous strains for a relatively short time, ranging from months to a few years [40,41]. Hence, further study of the immunology after norovirus infection and development of an approach to prolong the durability of the protective immunity after vaccination is important. Furthermore, reliable immune markers of norovirus immune protection need to be clearly defined. Current data suggest that mucosal IgA is a key factor to protect hosts from norovirus infection [30,42,43,44], implying that the adenovirus vectored Vaxart vaccine that is designed to induce the mucosal IgA response locally in the intestine [32] can be a good approach for high vaccine efficacy. Several human challenge investigations indicated that serum IgA, memory B cell responses, and serum HBGA-blocking titers are also immune correlates of protection [24,30,42,45]. By contrast, pre-existed serum IgG antibody levels seem not to play a protection role [35]. An epidemiological study even implied that serum norovirus-specific IgG titers were inversely related with protection against norovirus AGE [46], although information of norovirus genetic background and HBGA-related host susceptibility should also be taken into account in this context in future studies. Thus, it is essential to define and establish immunological correlates to truly indicate the protection of a norovirus vaccine. Based on our current understanding, a vaccine candidate that can stimulate broader immune responses to activate several lines of defense, including serum IgA, memory B-cell responses, and serum HBGA-blocking titers, will likely be a potent one. Finally, the lack of a cell culture-based neutralization assay and an effective animal model as tools to evaluate a norovirus vaccine represents other two challenges. In this context, the recent successes in the establishment of the human enteroid system [47] and the zebrafish larvae model [48,49] for norovirus replication have laid a solid foundation for future development of a conventional cell culture system and an effective animal model for noroviruses as useful tools to evaluate norovirus vaccines.

Norovirus infections induce both humoral and cellular immune responses. While antibody-mediated immunity is relatively well studied, our understanding of cellular immune response to human norovirus infection or vaccination remains limited [50,51]. As a result, most current clinical evaluations of norovirus vaccine candidates focus on the antibody-mediated immunity. A recent study [52] showed that GII.2 infection elicited broad antibody and cellular immunity activation for T cells, monocytes, and dendritic cells, offering a new paradigm and a future direction for norovirus vaccine development for broad immune response and protection.

In addition, the adjuvant is an important component of a successful vaccine to enhance the magnitude, breadth, and durability of the vaccine antigens. The currently tested adjuvants for norovirus vaccine candidates under clinical trials include aluminum salt for intramuscular delivery and a combination of chitosan and MPLA for intranasal administration of the VLP-based vaccines, as well as double-stranded RNAs for the adenovirus expressing VP1s. For future development, other adjuvants, particularly those that are licensed for use in human vaccines, including the oil-in-water emulsion adjuvant MF59, the Adjuvant Systems AS0 adjuvants, such as AS01, AS03, and AS04, and cytosine phosphoguanosine (CpG) 1018 (reviewed in [53]) should be tested for their potential to further improve the immunogenicity and protective efficacy of norovirus vaccines.

Finally, the current success of the mRNA vaccine platform in development of COVID-19 (coronavirus disease 2019) vaccines with high protective efficacy, as well as its further applications in developing vaccine candidates against influenza [54] and malaria [55], provide a new approach to advance the norovirus vaccine development program. Following the footsteps of COVID-19 vaccine development, noroviral VP1 or even its receptor binding protruding (P) domain that appears equivalent to the spike protein of SARS-CoV-2 (severe acute respiratory syndrome coronavirus 2) should be a good target of an mRNA vaccine against noroviral infection.

## Figures and Tables

**Table 1:** The four norovirus vaccine candidates under clinical evaluations *.

Company	Vaccine Candidate	Adjuvant	Administration Route	Antigen Format	Antigen Genotype	Status of Trial
Takeda	TAK-214	Chitosan/MPLA, aluminum salt	Intranasal, intramuscular	Noroviral VLP	GI.1/GII.4	Phase 2b clinical trial
Vaxart	VXA-NVV-104	Adenovirus expressing double-stranded RNAs	Oral	Adenovirus expressing noroviral VP1	GI.1/GII.4	Phase 1 clinical trial
NVSI	Hansenulapolymorpha	Aluminum salt	Intramuscular	Noroviral VLP	GI.1/GII.4	Phase 1 clinical trial
IPS/Zhifei	Longkoma	Aluminum salt	Intramuscular	Noroviral VLP	GI.1/GII.3/ GII.4/GII.17	Phase 2a clinical trial

(* see the main text for the abbreviations and other details).

## References

[1] Gaythorpe K.A.M., Trotter C.L., Lopman B., Steele M., Conlan A.J.K. (2018). Norovirus transmission dynamics: A modelling review. Epidemiol. Infect..

[2] Hall A.J., Glass R.I., Parashar U.D. (2016). New insights into the global burden of noroviruses and opportunities for prevention. Expert Rev. Vaccines.

[3] Hall A.J., Lopman B.A., Payne D.C., Patel M.M., Gastanaduy P.A., Vinje J., Parashar U.D. (2013). Norovirus disease in the United States. Emerg Infect. Dis..

[4] Mullaney S.B., Hyatt D.R., Salman M.D., Rao S., McCluskey B.J. (2019). Estimate of the annual burden of foodborne illness in nondeployed active duty US Army Service Members: Five major pathogens, 2010–2015. Epidemiol. Infect..

[5] GBD 2013 Mortality and Causes of Death Collaborators (2015). Global, regional, and national age-sex specific all-cause and cause-specific mortality for 240 causes of death, 1990–2013: A systematic analysis for the Global Burden of Disease Study 2013. Lancet.

[6] Bartsch S.M., Lopman B.A., Ozawa S., Hall A.J., Lee B.Y. (2016). Global Economic Burden of Norovirus Gastroenteritis. PLoS ONE.

[7] Giersing B.K., Vekemans J., Nava S., Kaslow D.C., Moorthy V. (2019). Report from the World Health Organization’s third Product Development for Vaccines Advisory Committee (PDVAC) meeting, Geneva, 8–10th June 2016. Vaccine.

[8] Chhabra P., de Graaf M., Parra G.I., Chan M.C., Green K., Martella V., Wang Q., White P.A., Katayama K., Vennema H. (2019). Updated classification of norovirus genogroups and genotypes. J. Gen. Virol..

[9] Van Beek J., Ambert-Balay K., Botteldoorn N., Eden J.S., Fonager J., Hewitt J., Iritani N., Kroneman A., Vennema H., Vinje J. (2013). Indications for worldwide increased norovirus activity associated with emergence of a new variant of genotype II.4, late 2012. Euro Surveill. Bull. Eur. sur les Mal. Transm. Eur. Commun. Dis. Bull..

[10] Van Beek J., de Graaf M., Al-Hello H., Allen D.J., Ambert-Balay K., Botteldoorn N., Brytting M., Buesa J., Cabrerizo M., Chan M. (2018). Molecular surveillance of norovirus, 2005–16: An epidemiological analysis of data collected from the NoroNet network. Lancet Infect. Dis..

[11] Siebenga J.J., Vennema H., Renckens B., de Bruin E., van der Veer B., Siezen R.J., Koopmans M. (2007). Epochal evolution of GGII.4 norovirus capsid proteins from 1995 to 2006. J. Virol..

[12] Seo H., Duan Q., Zhang W. (2020). Vaccines against gastroenteritis, current progress and challenges. Gut Microbes.

[13] Cates J.E., Vinje J., Parashar U., Hall A.J. (2020). Recent advances in human norovirus research and implications for candidate vaccines. Expert Rev. Vaccines.

[14] Esposito S., Principi N. (2020). Norovirus Vaccine: Priorities for Future Research and Development. Front. Immunol..

[15] Hallowell B.D., Parashar U.D., Hall A.J. (2019). Epidemiologic challenges in norovirus vaccine development. Hum. Vaccines Immunother..

[16] Mattison C.P., Cardemil C.V., Hall A.J. (2018). Progress on norovirus vaccine research: Public health considerations and future directions. Expert Rev. Vaccines.

[17] Cortes-Penfield N.W., Ramani S., Estes M.K., Atmar R.L. (2017). Prospects and Challenges in the Development of a Norovirus Vaccine. Clin. Ther..

[18] Baehner F., Bogaerts H., Goodwin R. (2016). Vaccines against norovirus: State of the art trials in children and adults. Clin. Microbiol. Infect..

[19] Lopman B.A., Steele D., Kirkwood C.D., Parashar U.D. (2016). The Vast and Varied Global Burden of Norovirus: Prospects for Prevention and Control. PLoS Med..

[20] Treanor J.J., Atmar R.L., Frey S.E., Gormley R., Chen W.H., Ferreira J., Goodwin R., Borkowski A., Clemens R., Mendelman P.M. (2014). A Novel Intramuscular Bivalent Norovirus Virus-Like Particle Vaccine Candidate-Reactogenicity, Safety, and Immunogenicity in a Phase 1 Trial in Healthy Adults. J. Infect. Dis..

[21] Atmar R.L., Baehner F., Cramer J.P., Song E., Borkowski A., Mendelman P.M., Al-Ibrahim M.S., Bernstein D.L., Brandon D.M., Chu L. (2016). Rapid Responses to 2 Virus-Like Particle Norovirus Vaccine Candidate Formulations in Healthy Adults: A Randomized Controlled Trial. J. Infect. Dis..

[22] Leroux-Roels G., Cramer J.P., Mendelman P.M., Sherwood J., Clemens R., Aerssens A., De Coster I., Borkowski A., Baehner F., Van Damme P. (2018). Safety and Immunogenicity of Different Formulations of Norovirus Vaccine Candidate in Healthy Adults: A Randomized, Controlled, Double-Blind Clinical Trial. J. Infect. Dis..

[23] Atmar R.L., Baehner F., Cramer J.P., Lloyd E., Sherwood J., Borkowski A., Mendelman P.M., Al-Ibrahim M.S., Bernstein D.L., Brandon D.M. (2019). Persistence of Antibodies to 2 Virus-Like Particle Norovirus Vaccine Candidate Formulations in Healthy Adults: 1-Year Follow-up With Memory Probe Vaccination. J. Infect. Dis..

[24] Atmar R.L., Bernstein D.I., Harro C.D., Al-Ibrahim M.S., Chen W.H., Ferreira J., Estes M.K., Graham D.Y., Opekun A.R., Richardson C. (2011). Norovirus vaccine against experimental human Norwalk Virus illness. N. Engl. J. Med..

[25] Bernstein D.I., Atmar R.L., Lyon G.M., Treanor J.J., Chen W.H., Jiang X., Vinje J., Gregoricus N., Frenck R.W., Moe C.L. (2015). Norovirus vaccine against experimental human GII.4 virus illness: A challenge study in healthy adults. J. Infect. Dis..

[26] Sherwood J., Mendelman P.M., Lloyd E., Liu M., Boslego J., Borkowski A., Jackson A., Faix D. (2020). Efficacy of an intramuscular bivalent norovirus GI.1/GII.4 virus-like particle vaccine candidate in healthy US adults. Vaccine.

[27] Tan M., Jiang X. (2005). The p domain of norovirus capsid protein forms a subviral particle that binds to histo-blood group antigen receptors. J. Virol..

[28] Tan M., Jiang X. (2010). Norovirus gastroenteritis, carbohydrate receptors, and animal models. PLoS Pathog..

[29] Tan M., Jiang X. (2011). Norovirus-host interaction: Multi-selections by human histo-blood group antigens. Trends Microbiol..

[30] Reeck A., Kavanagh O., Estes M.K., Opekun A.R., Gilger M.A., Graham D.Y., Atmar R.L. (2010). Serological correlate of protection against norovirus-induced gastroenteritis. J. Infect. Dis..

[31] Atmar R.L., Bernstein D.I., Lyon G.M., Treanor J.J., Al-Ibrahim M.S., Graham D.Y., Vinje J., Jiang X., Gregoricus N., Frenck R.W. (2015). Serological Correlates of Protection against a GII.4 Norovirus. Clin. Vaccine Immunol. CVI.

[32] AVaxart (2021). Vaxart Announces First Subject Enrolled in Phase 1b Norovirus Dose-Ranging Trial in Elderly Adults. https://investors.vaxart.com/news-releases/news-release-details/vaxart-announces-first-subject-enrolled-phase-1b-norovirus-dose.

[33] Kim L., Liebowitz D., Lin K., Kasparek K., Pasetti M.F., Garg S.J., Gottlieb K., Trager G., Tucker S.N. (2018). Safety and immunogenicity of an oral tablet norovirus vaccine, a phase I randomized, placebo-controlled trial. JCI Insight.

[34] Scallan C.D., Tingley D.W., Lindbloom J.D., Toomey J.S., Tucker S.N. (2013). An adenovirus-based vaccine with a double-stranded RNA adjuvant protects mice and ferrets against H5N1 avian influenza in oral delivery models. Clin. Vaccine Immunol. CVI.

[35] Johnson P.C., Mathewson J.J., DuPont H.L., Greenberg H.B. (1990). Multiple-challenge study of host susceptibility to Norwalk gastroenteritis in US adults. J. Infect. Dis..

[36] Lindesmith L.C., Ferris M.T., Mullan C.W., Ferreira J., Debbink K., Swanstrom J., Richardson C., Goodwin R.R., Baehner F., Mendelman P.M. (2015). Broad blockade antibody responses in human volunteers after immunization with a multivalent norovirus VLP candidate vaccine: Immunological analyses from a phase I clinical trial. PLoS Med..

[37] Zhou H., Chen L., Wang S., Tan M., Qiu C., Qiu T., Wang X. (2021). Prevalence and Evolution of Noroviruses between 1966 and 2019, Implications for Vaccine Design. Pathogens.

[38] Jin M., Wu S., Kong X., Xie H., Fu J., He Y., Feng W., Liu N., Li J., Rainey J.J. (2020). Norovirus Outbreak Surveillance, China, 2016–2018. Emerg Infect. Dis..

[39] Ao Y., Cong X., Jin M., Sun X., Wei X., Wang J., Zhang Q., Song J., Yu J., Cui J. (2018). Genetic Analysis of Reemerging GII.P16-GII.2 Noroviruses in 2016–2017 in China. J. Infect. Dis..

[40] Parrino T.A., Schreiber D.S., Trier J.S., Kapikian A.Z., Blacklow N.R. (1977). Clinical immunity in acute gastroenteritis caused by Norwalk agent. N. Engl. J. Med..

[41] O’Ryan M.L., Lucero Y., Prado V., Santolaya M.E., Rabello M., Solis Y., Berrios D., O’Ryan-Soriano M.A., Cortes H., Mamani N. (2009). Symptomatic and asymptomatic rotavirus and norovirus infections during infancy in a Chilean birth cohort. Pediatric Infect. Dis. J..

[42] Ramani S., Estes M.K., Atmar R.L. (2016). Correlates of Protection against Norovirus Infection and Disease-Where Are We Now, Where Do We Go?. PLoS Pathog..

[43] Costantini V.P., Cooper E.M., Hardaker H.L., Lee L.E., DeBess E.E., Cieslak P.R., Hall A.J., Vinje J. (2020). Humoral and Mucosal Immune Responses to Human Norovirus in the Elderly. J. Infect. Dis..

[44] Labayo H.K.M., Pajuelo M.J., Tohma K., Ford-Siltz L.A., Gilman R.H., Cabrera L., Mayta H., Sanchez G.J., Cornejo A.T., Bern C. (2020). Norovirus-specific immunoglobulin A in breast milk for protection against norovirus-associated diarrhea among infants. EClinicalMedicine.

[45] Lindesmith L., Moe C., Marionneau S., Ruvoen N., Jiang X., Lindblad L., Stewart P., LePendu J., Baric R. (2003). Human susceptibility and resistance to Norwalk virus infection. Nature Med..

[46] Ryder R.W., Singh N., Reeves W.C., Kapikian A.Z., Greenberg H.B., Sack R.B. (1985). Evidence of immunity induced by naturally acquired rotavirus and Norwalk virus infection on two remote Panamanian islands. J. Infect. Dis..

[47] Ettayebi K., Crawford S.E., Murakami K., Broughman J.R., Karandikar U., Tenge V.R., Neill F.H., Blutt S.E., Zeng X.L., Qu L. (2016). Replication of human noroviruses in stem cell-derived human enteroids. Science.

[48] Van Dycke J., Ny A., Conceicao-Neto N., Maes J., Hosmillo M., Cuvry A., Goodfellow I., Nogueira T.C., Verbeken E., Matthijnssens J. (2019). A robust human norovirus replication model in zebrafish larvae. PLoS Pathog..

[49] Van Dycke J., Cuvry A., Knickmann J., Ny A., Rakers S., Taube S., de Witte P., Neyts J., Rocha-Pereira J. (2021). Infection of zebrafish larvae with human norovirus and evaluation of the in vivo efficacy of small-molecule inhibitors. Nature Protoc..

[50] Lindesmith L., Moe C., Lependu J., Frelinger J.A., Treanor J., Baric R.S. (2005). Cellular and humoral immunity following Snow Mountain virus challenge. J. Virol..

[51] Lindesmith L.C., Donaldson E., Leon J., Moe C.L., Frelinger J.A., Johnston R.E., Weber D.J., Baric R.S. (2010). Heterotypic humoral and cellular immune responses following Norwalk virus infection. J. Virol..

[52] Lindesmith L.C., Brewer-Jensen P.D., Mallory M.L., Jensen K., Yount B.L., Costantini V., Collins M.H., Edwards C.E., Sheahan T.P., Vinje J. (2020). Virus-Host Interactions Between Nonsecretors and Human Norovirus. Cell. Mol. Gastroenterol. Hepatol..

[53] Pulendran B., Arunachalam P.S., O’Hagan D.T. (2021). Emerging concepts in the science of vaccine adjuvants. Nat. Rev. Drug Discov..

[54] Abbasi J. (2021). Pfizer Launches Phase 1 mRNA Flu Vaccine Trial. JAMA.

[55] Mallory K.L., Taylor J.A., Zou X., Waghela I.N., Schneider C.G., Sibilo M.Q., Punde N.M., Perazzo L.C., Savransky T., Sedegah M. (2021). Messenger RNA expressing PfCSP induces functional, protective immune responses against malaria in mice. NPJ Vaccines.

